# High-intensity versus low-level laser in musculoskeletal disorders

**DOI:** 10.1007/s10103-024-04111-1

**Published:** 2024-07-11

**Authors:** Marwa Shafiek Saleh, Mostafa Shahien, Hossam Mortada, Abdelrahman Elaraby, Yara Samir Hammad, Maged Hamed, Shorouk Elshennawy

**Affiliations:** 1 Basic Science Department, Faculty of Physical Therapy, Cairo University, Giza, Egypt; 2 Department of Physical Therapy, Faculty of Applied Medical Sciences, Al‐Zaytoonah University of Jordan, Amman, Jordan; 3 Faculty of Physical Therapy, Cairo University, Giza, Egypt; 4 Biomechanics Unit, Basic Sciences Department, Faculty of Physical Therapy, Galala University, Suez, Egypt; 5 Faculty of Physical Therapy, Misr University for Science and Technology, Giza, Egypt; 6 Department of Physical Therapy, Sharm El Shiekh International Hospital, South Sinai, Egypt; 7 Department of Pediatric Physical Therapy, Faculty of Physical Therapy, Cairo University, Giza, Egypt; 8 Department of Pediatric Physical Therapy, Faculty of Physical Therapy, Misr University for Science and Technology, Giza, Egypt

**Keywords:** HILT, LLLT, Laser, Low-level, High intensity, Musculoskeletal, Arthritis, Impingement, Carpal tunnel syndrome, Back pain

## Abstract

**Purpose:**

To evaluate the current evidence comparing low level to high level laser therapy to reveal any superiorities in the treatment of musculoskeletal disorders.

**Methods:**

Five databases were searched till September 2022 to obtain relevant RCTs comparing high intensity and low-level laser therapies in the management of musculoskeletal disorders. Two authors assessed the methodological quality of the included studies using the Physiotherapy Evidence Database scale and meta-analysis was conducted for studies that showed homogeneity.

**Results:**

Twelve articles were included in this systematic review with a total population of 704 participants across various musculoskeletal pathologies including tennis elbow, carpal tunnel syndrome, chronic non-specific low back pain, knee arthritis, plantar fasciitis, and subacromial impingement. There were no statistical differences between the two interventions in pain, electrophysiological parameters, level of disability, quality of life, postural sway or pressure algometer, however, Low level laser therapy showed superiority in increasing grip strength compared to high intensity laser therapy while results were significant in favour of high intensity laser therapy regarding long head of biceps diameter and cross sectional area, supraspinatus thickness and echogenicity and acromio-humeral distance.

**Conclusion:**

The current literature suggests no superiority of both types of laser therapy in musculoskeletal disorders, however, more RCTs with larger sample size are required to reach a definitive conclusion regarding the superiority of either form of laser therapy in musculoskeletal disorders.

## Introduction

Musculoskeletal disorders (MSD) are injuries and dysfunctions that have a negative impact on the human musculoskeletal system. They are the most common cause of chronic pain, physical functional impairment, and loss of quality of life [1, 2]. In addition, they are often the cause of an increased risk of other non-communicable diseases, such as heart disease [3].

Based on the latest analysis of the Global Burden of Disease data for 2019, there are globally about 1.71 billion people living with musculoskeletal diseases, including neck pain, low back pain, osteoarthritis, fractures, rheumatoid arthritis, and amputations [4]. Therefore, musculoskeletal problems are among the most significant contributors to the demand for rehabilitation therapy [4].

Different non-pharmacological interventions have been used in the management of MSD including acupuncture, exercise, manual therapy, and different physical therapy modalities [5]. One of the most important treatment methods that have been used as non-surgical treatments and painless methods for managing a wide range of MSD is laser therapy including low-level laser therapy (LLLT) and high-intensity laser therapy (HILT).

Low-intensity lasers can treat painful inflammatory conditions by lowering levels of biochemical markers, oxidative stress, neutrophil cell flow, and edema formation. It is therefore extensively used to treat acute and chronic painful conditions such as carpal tunnel syndrome, rheumatoid arthritis, knee inflammation, and injuries [6–8].

Low-intensity lasers can also alleviate pain by stimulating endorphin release and altering nerve excitation and conduction in peripheral nerves [9]. Besides its ability to regulate the functional characteristics of affected areas by promoting microcirculation and accelerating lateral circulation [10]. In addition, LLLT stimulates fibroblast proliferation and collagen synthesis which facilitate the healing process of tendon tissue after injury [11, 12].

Another form of laser therapy for controlling MSD is HILT, which is a novel form of laser therapy that has just emerged with unique properties such as an emission wavelength of 1,064 nm and a maximum output power of 3,000 W [13]. The high-intensity laser’s wavelength allows it to work with more focused and intense light energy, with a further increase in the concentration of endogenous chromophores during the treatment program.

In addition to its ability to diffuse more in tissues and for a deeper distance, it creates ATP and RNA, promotes the oxidative response to mitochondria, improves photo-biological effects on damaged tissues, and stimulates collagen synthesis in muscle tendons [14]. It has been reported that HILT applications have a significant effect on the recovery of nerve paralysis [13], wound repair [15], and pain relief [14].

Previous studies examined the separate effects of LLLT and HILT in the management of musculoskeletal disorders [16, 17]. However, based on the authors’ knowledge, no previous systematic review has examined the effect of HILT versus low LLLT in the management of musculoskeletal disorders. Therefore, this systematic review and meta-analysis aim to fill this gap and provide clear evidence regarding the superiority of either HILT or LLLT in clinical practice.

## Methods

This systematic review was registered in September 2022 in PROSPERO database record number CRD42022360797 and the authors adhered to the Preferred Reporting Items for Systematic Reviews and Meta-Analysis (PRISMA 2020) [18].

### Information sources and search strategy

A systematic search was conducted across five databases from inception to September 2022 searching for randomized controlled trials of a population suffering from any musculoskeletal disorders such as low back pain, knee osteoarthritis, plantar fasciitis, or elbow lateral epicondylitis. Participants received HILT compared to LLLT and outcome measures included any motor or sensory outcomes. Articles that have not been peer-reviewed and non-English written articles were excluded from this review Appendix 1.

### Study selection

The initial search results were imported and went through duplicate checking using Mendeley software. Two researchers independently screened titles and abstracts of the included articles using Rayyan web-based tool [19]. Full-text filtration process was conducted via another two authors. The senior author resolved any conflict in the filtration process.

### Quality assessment

The Physiotherapy Evidence Database (PEDro) scale [20] was used to assess the methodological quality of the included studies, the process was conducted by two authors independently. The PEDro Scale consists of 11 criteria where only ten criteria were calculated in the total score. The scored articles were considered to be of high quality, good quality, or fair quality if the score was from 9 to 10, 6–8, or from 4 to 5 respectively. The senior author was consulted in case of disagreements in the quality assessment process.

### Data collection process

The studies’ population and administered intervention data were extracted by two authors. Also, the results, outcome measures and domains were summarized into the data extraction sheet.

### Data synthesis

We conducted the meta-analysis through Cochrane Collaboration’s software Review Manager (RevMan version 5.3, Copenhagen). The post treatment scores were obtained in terms of mean and standard deviation for the pooled estimate. Data were combined as standardized mean differences (SMD) with 95% confidence intervals (CI). To solve the heterogeneity between included studies, I^2^ statistic was used. A percentage of ≥ 75% resembles considerable heterogeneity while a percentage of ≤ 40% reflects no heterogeneity of importance. A narrative description of the review finding will be elucidated if meta-analysis was not possible. The meta-analysis used the random-effect model, and its results were expressed as pooled effects, with corresponding 95% CIs and *P* values.

## Results

### Study selection

**Fig. 1:**
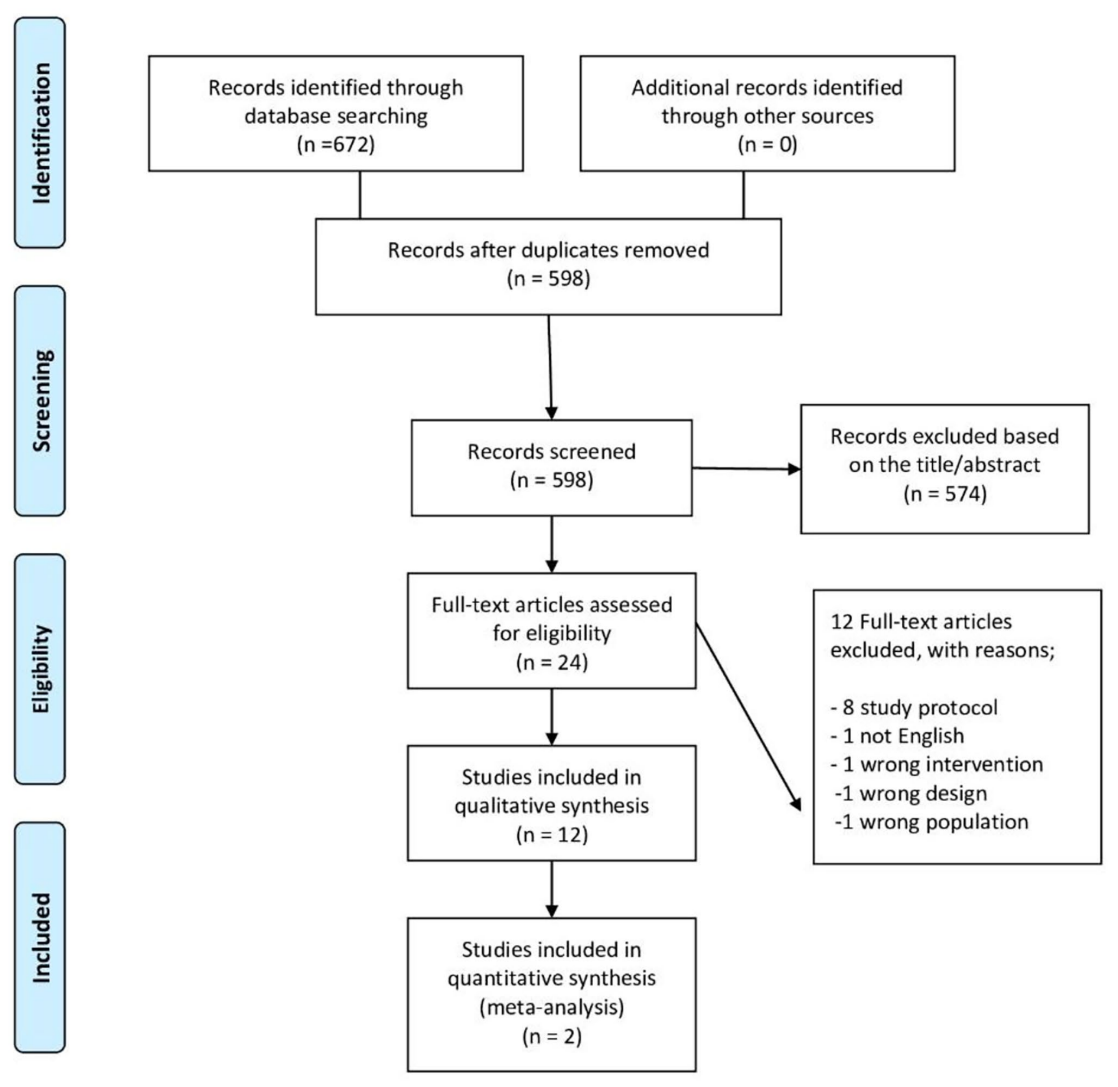
PRISMA flowchart of studies search and selection

Figure (1) shows the flow of the selection process throughout the study, an electronic search was conducted from inception till September 2022. The search study retrieved 56 records in PubMed, 74 in PEDro, 310 in Scopus, 116 in Google Scholar, 79 in Cochrane library, and 111 in Web of Science. After the removal of 74 duplicates, titles and abstracts of 598 study were screened. Twenty four studies underwent the phase of full text screening after the exclusion of 574. Initially, eight protocols were excluded because their full text papers have not been published yet. Three other studies were excluded due to the inappropriateness of intervention, population or design. The last study was excluded because it was not published in English.

### Quality appraisal

The twelve articles included were assessed with the PEDro scale. Two studies had a fair quality [21, 22], eight studies [23–30] deemed to be of a good quality, and the other two studies [31, 32] were assessed to be of an excellent quality. Throughout the 12 included studies, the main weakness points were regarding participants’, assessors’, and therapists’ blinding. Only half of the studies [21, 24, 25, 29, 31, 32] managed to blind the participants, while Ordahan et al. [30] was the only study that blinded the therapists.

### Characteristics of the included studies

We included a total of 12 articles, that fulfilled our inclusion criteria, and were described in Table (1). The studies’ publication date ranged from 2014 to 2021, with a total sample of 704 participants, and a range between 16 and 109 throughout the studies, with an average age range from 18 to 75. The RCTs included patients with various musculoskeletal pathologies; two studies [21, 32] included participants with tennis elbow disorders, three studies encompassed patients suffering from carpal tunnel syndrome, [23, 27, 31] two studies [25, 26] comprised people with chronic non-specific low back pain, two studies [22, 24] included participants with knee arthritis, two studies [29, 30] dealt with patients with unilateral plantar fasciitis, and the last study [28] included patients with subacromial impingement Table (2).

**Table 1:** Quality assessment scores

Study ID	Eligibility Criteria	Randomization	Allocation	Baseline Similarity	Subjects blinding	Therapists blinding	Assessor blinding	Dropout rate	Intention to treat analysis	Between groups comparison	Measure of variability	Total
Fekri et al., 2019	1	1	0	1	1	0	0	0	0	1	1	5
Hojjati et al., 2020	1	1	0	1	0	0	1	1	1	1	0	6
Kheshie et al., 2014	1	1	0	1	1	0	1	1	0	1	1	7
Taradaj et al., 2019	1	1	0	1	1	0	0	1	1	1	1	7
Abdelbasset et al., 2020	1	1	0	1	0	0	1	1	1	1	1	7
Sudiyono & Handoyo et al., 2020	1	1	0	1	0	0	1	1	0	1	1	6
Zaki et al., 2021	1	1	1	1	0	0	1	1	1	1	1	8
Ezzati et al., 2020	1	1	1	1	1	0	1	1	1	1	1	9
Kaydok et al., 2020	1	1	1	1	1	0	1	1	1	1	1	9
Kulchitskaya et al., 2015	0	1	0	1	0	0	0	0	0	1	1	4
Naruseviciute et al., 2020	1	1	1	1	1	0	0	1	0	1	1	7
Ordahan et al., 2018	1	1	1	1	0	1	1	1	0	1	1	8

**Table 2:** Population characteristics of the included studies

Study	Number	Gender	Age (Years)	Diagnosis	Post assessment	Follow up	Dropouts
Fekri et al.,2019	**30** G1(15), G2(15)	NR	32–67	Tennis elbow syndrome	After 10 sessions	-	No dropouts
Hojjati et al., 2020	**45** G1(15), G2(15), CG(15)	45 F	30–50	mild or moderate CTS	12 weeks	3 months	No dropouts
Sudiyono & Handoyo, 2020	**16** G1(8), G2(8)	15 F & 1 M	30–50	moderate CTS	2 weeks	-	No dropouts
Ezzati et al., 2020	**98** G1(20), G2(19), G3(20), G4(19), CG(20)	82 F & 16 M	20–60	CTS	3 weeks	-	2
Taradaj et al., 2019	**68** G1(18), G2(16), CG1(17), CG2(17)	36 M & 32 F	22–76	lumbar hernia disc & nonspecific chronic pain syndrome	3 weeks	1&3 months	No dropouts
Abdelbasset et al., 2020	**60** G1(20), G2(20), CG(20)	42 M & 18 F	25–40	Chronic nonspecific LBP	12 weeks	-	No dropouts
Kheshie et al., 2014	**53** G1(20), G2(18), CG(15)	53 M	54.6 ± 8.49	Knee OA	6 weeks	-	5
Zaki et al., 2021	**30** G1(10), G2(10), CG(10)	13 M & 17 F	40–60	Sub-acromial impingement	3 weeks	-	No dropouts
Kaydok et al., 2019	**59** G1(29), G2(30)	16 M & 43 F	18–65	Unilateral elbow pain	3 weeks	-	1
Naruseviciute et al., 2020	**109** G1(54), G2(55)	21 M & 81 F	18–85	Unilateral planter fasciitis	3 weeks	1 month	7 before post assessment 40 before follow-up
Ordahan et al., 2018	**75** G (38), G2(37)	15 M & 55 F	G1; 48.73 ± 11.41, G2; 48.65 ± 10.81	Unilateral planter fasciitis	3 weeks	-	5
Kulchitskaya et al., 2017	**60** G1(30), G2(30)	NR	40–75	Knee OA	After 10 sessions	12 months for G1	-

**CG**: Control group, **CTS**: Carpal tunnel syndrome, **F**: Female, **G**: Group, **LBP**: Low back pain, **M**: Male, **NR**: Not reported, **OA**: Osteoarthritis

The included papers vary according to intervention strategies with differences in the duration of application, wavelength, power and frequency. The total duration of the treatment varied from five sessions over two weeks [26] to 24 sessions over 12 weeks [31].

The power used for HILT ranged from 1.6 W [31] to 25 W [21], while that of the LLLT varied from 25 mw [27] to 800 mw [26]. Regarding the wavelength, it ranged between 808 nm [21] and 1064 nm [21, 22, 24, 25] for the HILT but concerning LILT, it was between 775 nm [23] and 905 nm [27]. The used frequency of the HILT was from 10 HZ [31] and 700 HZ [21], and the HILT was applied with a frequency that ranged from 10 HZ [31] to 6500 HZ [23] Table (3).

**Table 3:** Intervention characteristics of the included studies

Author ID	Study Groups	Control Groups	HILT Parameters	LLLT Parameters	Outcomes
Fekri et al., 2019	G1: HILT and traditional PT	None	- Average 3.3 and maximum 25 W power − 808 nm wavelength − 700 Hz frequency − 272.4 J energy - 13.4 J/cm density − 5 cm target area - 3 min duration 10 sessions, six days a week	− 100 mW power − 808 nm wavelength − 250 Hz frequency − 8 J/cm density − 4 min duration 10 sessions, six days a week	- Pain (VAS) - Tenderness (algometer) - Grip force (dynamometer)
	G2: LLLT and traditional PT				
Hojjati et al., 2020	G1: wrist splint + HILT	Wrist splint only	-5 W power − 1064 nm wavelength -20 J/cm^2^ density − 36 s 12 weeks	− 45 W power − 775 nm wavelength − 20 J/cm^2^ density 6500 Hz frequency 12 weeks	- Pain (VAS) - Function (Boston questionnaire) - Pinch strength (dynamometer) - SNAP amplitude and PL - CMAP OL, amplitude and NCV
	G2: wrist splint + LLLT				
Sudiyono & Handoyo, 2020	HILT	None	− 12 W power − 1064 nm wavelength − 10 J/cm^2^ (analgesic)/ 120 J/cm^2^ (biostimulation) density Five session / week for two weeks	− 25 mW power − 905 nm wavelength − 6 J/cm^2^ density Five session / week for two weeks	- Combined sensory index - Sensory nerve conduction velocity - Distal motoric latency
	LLLT				
Ezzati et al., 2020	G1,2: Exercise therapy and LLLT	Exercise therapy only	− 1.6 W average power − 808 nm wavelength − 10 HZ frequency − 8 (G3)/ 20 (G4) J/cm^2^ density − 100/250 sec duration per point with total 10 points Five sessions / week for two weeks	− 50 mW power − 860 nm wavelength − 8 (G1) /20 (G2) J/cm^2^ density − 32/76 sec duration per point with total 10 points Five sessions / week for two weeks	- Pain (VAS) - CMAP latency - CMAP amplitude - CMAP conduction velocity - SNAP latency - SNAP amplitude
	G3,4: Exercise therapy and HILT				
Taradaj et al., 2019	G1: HILT	CG1: sham HILT	− 1064 nm wavelength − 30 cm^2^ area − 60 J/cm^2^ density − 10 min duration Five sessions / week for three weeks	− 785 nm wavelength − 30 cm^2^ area − 8 J/cm^2^ density − 8 min duration Five sessions / week for three weeks	Postural control parameters as follows: - Total sway path (open and closed eyes) - Sway path along Y and X-axes (open and closed eyes) - Mean velocity of COP displacement (open and closed eyes) - Mean sway frequency (open and closed eyes) - Sway area
	G2: LLLT	CG2: sham LILT			
Abdelbasset et al., 2020	LLLT + home exercise training	Home exercise training only	− 12 W power − 1064 nm wavelength − 150 J/cm^2^ density − 1200 J total energy − 15 min per session Two sessions / week for 12 weeks	− 800 mw power − 850 nm wavelength − 1 kHz frequency with 80% duty cycle − 50 J/cm^2^ density − 1200 J total energy − 30 min per session Two sessions / week for 12 weeks	- Disability level (ODI) - Pain (VAS) - Lumbar Flexion ROM - Quality of life (EuroQol)
	HILT + home exercise training				
Kheshie et al., 2014	G1: HILT and Exercises.	Placebo + Exercises	− 610, 710, 810 mJ/cm^2^ density − 1250 J total energy − 15 min duration per session Two session / week for six weeks	− 800 mW power - 830 nm wavelength - 50 J/cm^2^ density - 1 kHz frequency with 80% duty cycle - 32 min and 33 s duration per session Two session / week for six weeks	- Pain (VAS) - Knee and lower limb functions (WOMAC)
	G2: LLLT and Exercises				
Zaki et al., 2021	G1: LLLT + KT	Sham laser and KT	− 4000 mw power - 810 and 980 nm wavelengths − 20.5 J/cm^2^ density − 513 s per session duration − 2050 J total energy seven sessions, three a week	− 200 mw power − 810 and 980 nm wavelengths - 20 J/cm^2^ density − 1000 s per session duration − 2000 J total energy seven sessions, three a week	- Pain (VAS and SPADI) - Shoulder pain and disability index - Musculoskeletal ultrasound imaging
	G2: HILT + KT				
Kaydok et al., 2019	G1: HILT	None	− 1064 nm wavelength 1st phase (analgesic): - 8 W power - 6 J/cm^2^ density - 150 J total energy - 75 s per point - Three sessions for one weeks 2nd phase (biostimulation): - 6 W power - 120:150 J/cm^2^ density - 30 s per point - Three sessions per week for two weeks	− 240 mW power - 904 nm wavelength - 5000 Hz frequency - 2.4 J/cm^2^ density - 30 s per point Three sessions per week for three weeks	- Pain (VAS) - Hand function (QDASH) - Hand grip strength (dynamometer) - Quality of life (SF-36)
	G2: LLLT				
Naruseviciute et al., 2020	G1: LLLT + PE	None	− 7 W power - 1064 nm wavelength - 120 J/cm^2^ density - 3000 J total energy - 7 min and 8 s duration Three session / week, for a total of eight sessions	− 50 mW power - 785 nm wavelength - 4 J/cm^2^ density - 140 J total energy - 6 min and 40 s duration Three session / week, for a total of eight sessions	- Pain (VAS) - Tenderness (algometer) - Plantar fascia thickness (sonography)
	G2: HILT + PE				
Ordahan et al., 2018	G1: HILT	None	− 1064 nm wavelength 1st phase (analgesic): - 8 W power - 6 J/cm^2^ density - 150 J total energy - 75 s - Three sessions for one weeks 2nd phase (biostimulation): - 6 W power - 120:150 J/cm^2^ density - 30 s - Three sessions per week for two weeks	− 240 mW power - 904 nm wavelength - 5000 Hz frequency - 8.4 J/cm^2^ density - 157.5 s per session Three sessions per week for three weeks	- Function and quality of life (FAOS) - Pain (VAS and HTI)
	G2: LLLT				
Kulchitskaya et al. 2017	G1: HILT	None	− 1064 nm wavelength - 25 Hz frequency - 10 J/cm^2^ density - 4 min duration 10 sessions every alternate day	− 890 nm wavelength - 80 Hz frequency - 40 W power - 4 min duration 10 sessions every alternate day	State of the capillary blood flow (LDF)
	G2: LLLT				

**CMAP**: Compound muscle action potential, **COP**: Center of pressure, **FAOS**: Foot and Ankle Outcome Score, **G**: Group, **HILT**: High Intensity Laser Therapy, **HTI**: Heel tenderness index, **KT**: Kinesiotape, **LDF**: Laser Doppler Flowmetry, **LLLT**: Low level laser therapy, **Min**: Minutes, **NCV**: Nerve conduction velocity, **ODI**: Oswestry disability index, **OL**: Motor onset latency, **PL**: Peak latency, **PT**: Physiotherapy, **QDASH**: Quick disabilities of arm, shoulder and hand, **Sec**: Seconds, **SF-36**: 36-item short form survey, **SNAP**: Sensory nerve action potential, **SPADI**: Shoulder pain and disability index, **VAS**: Visual analogue scale, **WOMAC**: Western Ontario and McMaster Universities Osteoarthritis Index

### Outcome measures

#### Pain

**Fig. 2:**
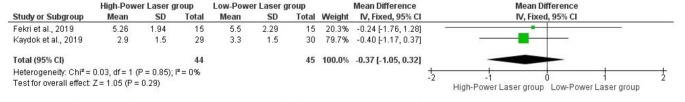
Forest plot of comparison: between HILT and LLLT, outcome pain

Nine studies [21, 23, 24, 26, 28–32] examined the efficacy of HILT vs. LLLT in reducing pain using VAS in patients with subacromial impingement, carpal tunnel syndrome, knee osteoarthritis, plantar fasciitis and lateral elbow pain, and they reported no significance difference between both groups except for kheshie et al. [24] which reported a significant difference for the HILT group. Additionally, one study [30] used heel tenderness index to assess pain among patients with plantar fasciitis without revealing different results. These results are supported by a quantitative analysis for two studies [21, 32] which showed a non- statistical significance difference between both interventions, SMD − 0.37 (-1.05,0.32, *P* = 0.29) Fig. (2).

#### Hand grip strength

**Fig. 3:**
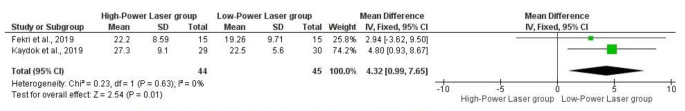
Forest plot of comparison: between HILT and LLLT, outcome grip strength

Based on a quantitative analysis, two studies [21, 32] showed that LLLT is favourable over HILT in improving grip strength measured by dynamometer SMD,4,32 (0.99,7.65, *P* = 0.01) Fig. (3).

#### Ultra-sonographic parameters

Two studies [28, 29] measured long head of biceps diameter and cross sectional area, supraspinatus thickness and echogenicity, acromio-humeral distance which were significant in favour of HLLT, however, plantar fascia thickness was not significant between groups.

#### Electrophysiological parameters

Three studies [23, 27, 31] measured respectively sensory nerve action potential (SNAP), compound muscle action potential (CMAP), SNAP peak latency and amplitude, CMAP onset latency and amplitude, combined sensory index, sensory nerve conduction velocity, distal motor latency revealing no significant between HILT and LLLT.

#### Disability scales

Several measurement scales were used in order to demonstrate the level of disability using the following, shoulder pain and disability index for subacromial impingement [28], quick disability of arm, shoulder and hand for lateral epicondylitis [32], western Ontario and McMaster universities osteoarthritis index for knee osteoarthritis (WOMAC) [24], oswestry disability index for low back pain [26], foot and ankle outcome scales for plantar fasciitis [30] and boston symptoms severity scale for carpal tunnel syndrome [23]. However, all of them reported non-significant differences between both interventions except for the WOMAC scale which was significant for the HILT group.

#### Quality of life scales

Two measurement scales short form health survey (SF-36) [32] and EuroQol [26] were used to evaluate the efficacy of HILT versus LLLT in improving life quality in patients with lateral epicondylitis and low back pain respectively, and both results were not significant between both groups.

#### Postural sway parameters

One study [25] compared HILT versus LLLT in improving postural control parameters in patients with chronic non-specific low back pain, and it showed no significance between both interventions.

#### Algometer measures

Two studies [21, 30] used the pressure algometer device to evaluate the efficacy of both interventions in enhancing the tenderness threshold in patients with lateral epicondylitis and plantar fasciitis. According to their reports, there was no advantage for any of the administered interventions.

## Discussion

This systematic review of randomized controlled trial revealed LLLT has a significant effect on grip strength of patients with lateral epicondylitis compared to HILT however no statistically significant difference between HILT and LLLT in terms of musculoskeletal pain reduction while the results were conflicting concerning level of disability, quality of life and electrophysiological parameters such as the amplitude and latency of both CMAP and SNAP obtained from patients with carpal tunnel syndrome.

The improvement of grip strength in the LLLT group may be attributed to the increase of ATP, collagen production and the facilitation of healing induced by LLLT which in turn increase the designated muscles’ power and grip strength [33].

The results of no superiority of any of the investigated intervention in terms of pain contradict with the previous results reported by Kheshie et al., 2014 which demonstrated significant effects of HILT in pain reduction compared to LLLT, this may be due to the different population investigated i.e., patients with knee osteoarthritis and the ability of the HILT to penetrate deeply in the anatomical configuration of the treated joint.

Regarding electrophysiological parameters, Ezzati et al., 2020 [31] which measured CMAP amplitude and latency and SNAP amplitude and latency of the median nerve groups in patients with carpal tunnel syndrome reported a significant effect in favor of the HILT. However, the other two studies conducted on the same targeted population [23, 27] were not significant. This may be due to the larger population i.e., 98 participants and the longer duration of treatment in Ezzati et al., 2020 that exceeded the other two studies with a range of 100 to 250 s.

Moving to the level of disability, only two studies [24, 32] out of five revealed significant difference between the two types of laser therapy in favour of HILT however, this difference was marginal in one of them (*p* = 0.046) while the improvement of level of disability in the other study may be linked to the pain reduction in the HILT group compared to the LLLT, hence, the level of disability would probably decrease more than the other group.

Similarly, the quality of life scores of the same study [32] were significant regarding physical component which may be linked to the improvement of grip strength in such population i.e., patients with lateral epicondylitis. In contrast, the quality of life in patients with chronic low back pain was not significant as the pain level did not show any difference [26].

Compared to previously reported results, LLLT and HILT are considered as effective modalities in reducing musculoskeletal pain [16, 17]. Furthermore, both modalities are effective in the management of knee osteoarthritis when it comes to knee pain and function outcomes [34]. However, due to the originality of the research question, no previous systematic review examined the comparative efficacy of both interventions, therefore, reported results cannot be compared with other studies.

A number of limitations could be noted in this review. Due to the novelty of our idea, we could not compare our results with other reviews as it is the first one which evaluates the comparative efficacy of HILT versus LLLT among individuals with musculoskeletal disorders. Furthermore, limited number of randomized clinical trials were found which compared the efficacy of HILT to LLLT in among musculoskeletal diseases, and this limited the ability to formulate a strong evidence to prefer one of the both interventions over the other in addition to the applicability of performing meta-analysis which was only possible in only two studies sharing the same characteristics as the rest of the studies were heterogeneous regarding the population and the measured outcomes.

## Conclusion

There is insufficient evidence concerning the superiority of HILT therapy over LLLT in patients with musculoskeletal diseases due to the lack of large number of randomized controlled trials with adequate sample size. However, both interventions are considered as safe and valuable tools as no side effects have been detected with the application of both interventions, and they could be implemented in the rehabilitation programs to promote the efficacy of our treatment plans.

## Data Availability

All the data used in the current article is either reported in the manuscript or available in the included studies reports.
